# Patient-Reported Compliance in older age patients with chronic heart failure

**DOI:** 10.1371/journal.pone.0231076

**Published:** 2020-04-16

**Authors:** Beata Jankowska-Polańska, Natalia Świątoniowska-Lonc, Agnieszka Sławuta, Dorota Krówczyńska, Krzysztof Dudek, Grzegorz Mazur

**Affiliations:** 1 Department of Clinical Nursing, Faculty of Health Science, Wroclaw Medical University, Wroclaw, Poland; 2 Department of Internal Diseases, Occupational Medicine, Hypertension and Clinical Oncology, Wroclaw Medical University, Wroclaw, Poland; 3 Medical University of Warsaw, Department of Clinical Nursing, Warsaw, Poland; 4 Faculty of Mechanical Engineering, Technical University of Wroclaw, Wroclaw, Poland; 5 Department of Internal Diseases, Occupational Medicine, Hypertension and Clinical Oncology, Wroclaw Medical University, Wroclaw, Poland; Maastricht University Medical Center, NETHERLANDS

## Abstract

The purpose of the study was to evaluate compliance with medical treatment in elderly patients with heart failure (HF), and to identify factors that associated with patients’ compliance levels.

**Methods and results:**

475 patients (including 222 women), mean age 69.7±7.7, with HF, hospitalized at University Hospital between January and December 2018 were included in the study. The patients were selected by a physician specializing in cardiology. A cardiac nurse assessed the non-pharmacological level of compliance using the Revised Heart Failure Compliance Questionnaire (RHFCQ). The socio-clinical data were obtained from medical records. The majority of the study group were patients in NYHA II (62.4%) and NYHA III (28.3%), the mean duration of the disease was 6.2±4.9 years, and the mean ejection fraction of the left ventricle (EF) was 48.6±12.6. The average level of compliance in the study group measured on a scale from 0 to 4 points was: median = 2.7, IQR [2.32; 3.25]. Only 6.9% of the respondents adhere to recommendations totally (all dimensions of RHFCQ). In univariate analysis, predictors negatively affecting compliance were: female gender (rho = -0.325), age below 65 years (rho = -0.014)), loneliness (rho = -0.559), number of hospitalizations (rho = -0.242), higher stage of NYHA (rho = -1.612), co-morbidities (rho = -0.729), re-hospitalizations (rho = -0.729), beta-blockers treatment (rho = -1.612) and diuretics treatment (rho = -0.276). Factors positively affecting compliance were: EF≥45% (rho = 0.020) and treatment with ACEI/ARB (rho = 0.34), whereas compliance was negatively affected by–EF<45% (β = 0.009). Independent predictors influencing the level of compliance were: loneliness (β = -1.816), number of hospitalizations (β = -0.117), NYHA III and IV and number of co-morbidities (β = -0.676).

**Conclusions:**

Patients with HF do not adhere to therapeutic recommendations. The lowest compliance levels were found for exercise and daily weighing, and the highest for follow-up appointment-keeping and medication. Loneliness and age are the strongest predictors which influence the level of compliance.

## Introduction

Heart failure is the most common cause of hospitalization for patients older than 65. Despite developments in cardiovascular treatment, the high hospitalization rate has not changed for the last twenty years, and is currently one of the most significant challenges for health care systems worldwide [1]. The incidence and prevalence of heart failure increase strikingly with age and make heart failure the most common reason for hospitalization among older adults. Although outcomes for older adults with heart failure have improved over time, mortality, hospitalization, and re-hospitalization rates remain high. Over 80% of patients with HF are more than 65 years old, in addition they can be accompanied by other numerous diseases and clinical syndromes. Management of heart failure in older age remains a challenge. Epidemiological data show that following a first hospitalization for HF, 25% of patients are re-hospitalized within 30 days, and 50% are re-hospitalized within six months [2].

Chronic heart failure is treated both pharmacologically and non-pharmacologically. The guidelines underline the importance of non-pharmacological recommendations for patients with heart failure: restriction of sodium intake, reduction of fluids, early detection of deterioration by monitoring symptoms (including daily weighing), reduction of alcohol consumption, smoking cessation and maintenance of activity [1].

Compliance has been defined as the extent to which the behavior of a given person is in line with health recommendations [3]. Non-compliance to treatment may result from a variety of causes, associated with the underlying conditions leading to HF; the course of HF; patient characteristics, including education, awareness, involvement in the treatment process, beliefs about medication and social support; and also the specific treatment protocol used, including the availability of medication or its potential adverse effects [4–6]. In older age patients with chronic diseases and multiple-drug problems, different levels of willingness and ability to follow pharmacological recommendations are observed. It is highly probable that their attitude to treatment, convictions and fears may significantly influence the level of adjustment to the established therapeutic schemes. Older people may have developed beliefs and views about the drugs used, often based on their own or their family’s experience. In the case of patients with polypharmacy, the risk of side effects increases as the patients may have habits of abusing medical preparations or believe that the treatment used is of little benefit to them and may even be harmful [7]. In the literature, compliance rates to medication in older age patients vary from 10% to 99%. The prevalence of non-compliance increases with age [6].

Non-pharmacological treatment is additionally complicated by difficulties directly related to HF symptoms, such as dyspnea or excessive thirst. Non-compliance to non-pharmacological treatment recommendations (e.g. regular moderate exercise) [8] results in a gradual deterioration of the patient’s functional status, especially in terms of effort tolerance and muscle mass, and faster development of negative outcomes (cardiac cachexia), even with full compliance with other aspects of the treatment [9]. The literature shows that compliance with the sodium-restricted diet in older age patients with HF varies from 13% to 75%, fluid restriction 23–70%, daily weighing 12–79%, and exercise training 44–56% [6,10]. Medication is a critical part of HF treatment, and compliance with medication is a key aspect of HF self-care [11]. Sadly, treatment compliance is low in HF patients, leading to worse clinical outcomes and more HF exacerbations, poorer physical function, and a higher risk of hospitalization and death [4,7,11]. It is broadly known that the prognosis for patients can additionally be improved effectively by lifestyle modifications and optimized self-care. However, these are implemented in patients’ everyday lives to an unsatisfactory degree due to the lack of the acceptance as well as the lack of good cooperation between physician and patient.

The purpose of the study was to evaluate treatment compliance level (both pharmacological and non-pharmacological) in older age patients with HF, and to identify factors that affect patients’ compliance levels.

## Material and methods

A hospital-based cross-sectional study was conducted at University Hospital between January and December 2018.

Patients included in the study were 60 years old or above, diagnosed with HF, taking medications, and followed up for at least one month before the start of the study. Exclusion criteria were as follows: (1) requirement for intensive cardiac care, (2) inability to complete the questionnaires or lack of consent to participate in the study, (3) severe depression or terminal illness.

The study was approved by the local Bioethics Committee (No KB 67/2016). Patients were selected by a physician specializing in cardiology. Questionnaires were distributed by a cardiac nurse. All patients completed all questionnaires. Demographic and socio-demographic data (age, gender, years of education, marital status) were obtained from interviews performed by a cardiac nurse and from patient records. Clinical data, such as the New York Heart Association (NYHA) functional class, left ventricular ejection fraction (EF), number of re-hospitalizations, and the medication taken were obtained from records and from personal interviews with the participants performed by a cardiac nurse. Participation was voluntary and anonymous, and all patients were informed about its purpose and their right to decline or discontinue their participation. Written informed consent was obtained from each participant prior to the inclusion before the interview. The investigation conformed to the principles outlined in the Declaration of Helsinki. Patients who did not know how to take their medication or follow other instructions were counseled on these points at the end of the interview. Data confidentiality was assured by using assigned code numbers in lieu of participants’ names.

Compliance with recommendations regarding a sodium-restricted diet, fluid restriction, exercise, and daily weighing was measured using the Revised Heart Failure Compliance Questionnaire (RHFCQ). Internal consistency for the original version of instrument was tested using Cronbach α with the result of 0.68 [12]. The questionnaire measures compliance on a 6-item scale. Patients are asked to rate their compliance in the past week (medication, sodium-restricted diet, fluid restriction, and exercise), the past month (daily weighing), or the last 3 months (appointment keeping) before index hospitalization. Patients are considered to comply with a recommendation if they state they follow it “always” or “most of the time”. In terms of daily weighing, patients are considered compliant when they weigh themselves daily or ≥ 3 times a week. Patients are considered “overall compliant” if they comply with ≥ 4 out of the 6 recommendations. The questionnaire has good psychometric properties, with a Cronbach’s alpha of 0.768 for the group studied and average inter-item correlation of 0.362 [13].

### Statistical analysis

Depending on their distribution, quantitative variables are shown in tables and figures as means (M) and standard deviations (SD) or median (Me) and quartile (Q1 and Q3) values. Empirical distribution normality for quantitative variables was verified using the Shapiro-Wilk test.

The significance of differences between quantitative variable distributions in the groups was determined using the Mann-Whitney U-test. Cut-off values for continuous variables were determined by ROC curve analysis.

To counteract the problem of multiple comparisons, the tables present levels of significance of tests after applying Bonferroni or Dunn correction. Stepwise Multiple Regression was used to analyse the relationship between the dependent variable (compliance) and several independent variables (which were significant in univariate analysis). Because the regression coefficients have different measures (depending on the type of independent variables considered), estimates of the standard coefficients Beta are presented in the table as the result of the analysis. The variables included in the models were strongly correlated with the dependent variable and at the same time weakly correlated with each other.

The impact of quantitative and qualitative variables of compliance levels in the RHFCQ domains was analyzed using single- and multiple-factor regression. For all tests, p < 0.05 was used as a statistical significance threshold. Calculations were performed using STATISTICA v.12 software (StatSoft, Inc., USA).

## Results

### Socio-clinical characteristics of the HF patients studied

A total of 475 HF patients (included 222 female, in mean age of 69.7±7.7 years) met the inclusion criteria and were included in the study. The mean duration of illness was 6.2 ± 4.9 years. At the time of the interview, most respondents were classified in NYHA class II (62.8%) or III (28.3%), with a mean EF (Left ventricular ejection fraction) of 48.6 ± 12.2. The patients were hospitalized 2 ± 1.5 times a year, and were diagnosed with two chronic diseases in addition to HF (most commonly arterial hypertension). Most patients were treated for HF with diuretics (80.8%) and angiotensin-converting enzyme (ACE) inhibitors (82.6%). (Table 1).

**Table 1:** Socio-clinical characteristics of the HF patients studied.

Characteristic (variable)	Statistics
Gender:	
Female	46.7% (222)
Male	53.3% (253)
Age (years):	
*M ± SD*	69.7 ± 7.7
*Me* [*Q*1; *Q*3]	68 [64; 73]
Living alone	43.6% (206)
Professionally active	4.6% (22)
Duration of the illness (years):	
*M ± SD*	6.2 ± 4.9
*Me* [*Q*1; *Q*3]	5 [3; 8]
NYHA class:	
II	62.8% (298)
III	28.3% (135)
IV	8.8 (42)
EF:	
*M ± SD*	48.6 ± 12.6
*Me* [*Q*1; *Q*3]	50 [40; 60]
Number of hospitalizations:	
*M ± SD*	2.0 ± 1.5
*Me* [*Q*1; *Q*3]	1 [1; 3]
Number of comorbidities:	
*M ± SD*	1.8 ± 1.2
*Me* [*Q*1; *Q*3]	1 [1; 2]
Medication:	
Diuretics/aldosterone receptor antagonists	80.8% (384)
β-blockers	68.2% (324)
ACE inhibitors/angiotensin receptor blockers (ARBs)	85.5% (406)

### Compliance with HF treatment

Respondents obtained the highest HF treatment compliance scores in terms of follow-up appointment-keeping (3.3 ± 1.0) and taking medication as prescribed (3.3 ± 0.9), and the lowest in terms of regular weighing (0.9 ± 1.1) and exercise (1.1 ± 1.2). More than half of the respondents (57.5%) kept their follow-up appointments and took their medication regularly. Daily weighing was reported by as few as 11.8% of respondents, with slightly higher percentages reported limiting their sodium (17.1%) and fluid intake (13.9%). The fewest patients regularly exercised as ordered by their physician (3.6%) (Table 2).

**Table 2:** Adherence to HF treatment as measured by the RHFCQ questionnaire.

Responses to RHFCQ items	*M ± SD* *Me* [*Q*1; *Q*3] % (*n*)
1. Domain: follow-up appointment keeping; In the past three months, how often did you keep your follow-up appointments?	3.3 ± 1.0 4 [3; 4]
Never	1.3% (6)
Seldom	3.2% (15)
Half of the time	19.2% (91)
Most of the time	18.9% (90)
Always	57.5% (273)
2. Domain: taking medication as prescribed In the past week, how often did you take your medication?	3.3 ± 0.9 4 [3; 4]
Never	1.9% (9)
Seldom	0.6% (3)
Half of the time	17.9% (85)
Most of the time	22.1% (105)
Always	57.5% (273)
3. Domain: daily weighing In the past month, how often did you weigh yourself?	0.9 ± 1.1 0 [0; 2]
Every day	11.8% (56)
Three times a week	17.3% (82)
Once a week	17.3% (82)
Less than once a week	53.7% (255)
4. Domain: restricted sodium intake In the past week, how often did you keep a low-sodium diet?	2.1 ± 1.3 2 [1; 3]
Never	12.8% (61)
Seldom	21.9% (104)
Half of the time	25.9% (123)
Most of the time	22.3% (106)
Always	17.1% (81)
5. Domain: restricted fluid intake In the past week, how often did you limit your fluid intake?	1.8 ± 1.4 2 [1; 3]
Never	25.1% (119)
Seldom	17.3% (82)
Half of the time	21.7% (103)
Most of the time	22.1% (105)
Always	13.9% (66)
6. Domain: regular exercise In the past week, how often did you exercise as ordered by your physician?	1.1 ± 1.2 1.2 1 [0; 2]
Never	47.8% (227)
Seldom	19.4% (92)
Half of the time	15.8% (75)
Most of the time	13.5% (64)
Always	3.6% (17)

### The level of compliance according to RHFCQ–comparative analysis

Comparative analysis of compliance levels considering the selected socio-demographic variables demonstrated differences between men and women only for the domain “taking medication as prescribed”, with female patients scoring lower than male patients (3 vs. 4). However, significant differences in compliance were found for age and social status groups. Patients older than 65 and those living alone had lower compliance scores in all RHFCQ domains and lower overall compliance scores (Table 3).

**Table 3:** Statistics for scores (median, IQR and range) in each RHFCQ domain broken down by selected socio-demographic variables (gender, age, and social status).

Adherence to HF treatment (RHFCQ)	Gender		p-value	Age (years)		p-value	Living		*p*-value
	Female N = 222	Male N = 253		≤ 65 N = 158	> 65 N = 253		with someone N = 269	alone N = 152	
1. Follow-up appointment keeping	4 [2; 4]	4 [3; 4]	0.169	4 [3; 4]	4 [2; 4]	**0.006**	4 [3; 4]	3 [2; 4]	**<0.001**
	0–4	0–4		0–4	0–4		0–4	0–4	
2. Taking medication as prescribed	3 [2; 4]	4 [3; 4]	**<0.001**	4 [3; 4]	4 [3; 4]	**0.034**	4 [3; 4]	3 [2; 4]	**<0.001**
	0–4	0–4		0–4	0–4		0–4	0–4	
3. Daily weighing	0 [0; 2]	0 [0; 3]	0.593	0 [0; 2]	0 [0; 1]	**<0.001**	1 [0; 2]	0 [0; 1]	**0.001**
	0–3	0–3		0–3	0–3		0–3	0–3	
4. Restricted sodium intake	2 [1; 3]	2 [1; 3]	0.672	2 [2; 3]	2 [1; 3]	**0.004**	2 [1; 3]	2 [1; 3]	**<0.001**
	0–4	0–4		0–4	0–4		0–4	0–4	
5. Restricted fluid intake	2 [1; 3]	2 [0; 3]	0.485	2 [1; 3]	2 [0; 3]	**0.001**	2 [1; 3]	2 [0; 3]	**0.009**
	0–4	0–4		0–4	0–4		0–4	0–4	
6. Regular exercise	0 [0; 2]	1 [0; 2]	0.059	1 [0; 2]	0 [0; 2]	**<0.001**	1 [0; 2]	0 [0; 1]	**<0.001**
	0–4	0–4		0–4	0–4		0–4	0–4	
7. Healthy lifestyle	7 [3; 10]	7 [3; 10]	0.725	8 [4; 11]	6 [2; 10]	**<0.001**	8 [4; 11]	6 [2; 9]	**<0.001**
	0–15	0–15		0–15	0–14		0–15	0–15	
Overall adherence level	13 [9; 17]	13 [9; 17]	0.595	15 [12; 19]	12 [9; 16]	**<0.001**	15 [11; 18]	11 [8; 16]	**<0.001**
	4–23	1–23		1–23	4–22		1–23	4–22	

Median [IQR] and range

Comparative analysis of compliance levels considering selected clinical variables demonstrated statistically significant differences in compliance. Patients with EF below 45% had lower compliance scores in domains: “follow-up appointment keeping” (3 vs. 4), “taking medication as prescribed” (3 vs. 4), and “regular exercise”(0 vs. 1) than those with EF≥45%. Differences in compliance were also associated with the duration of the illness. Patients living with HF for less than 4 years had higher compliance scores than those with a longer duration of illness in the following domains: “daily weighing” (2 vs. 0), “restricted sodium intake” (2 vs. 1), “restricted fluid intake” (2 vs. 1), and “regular exercise” (1 vs. 0). Statistically significant differences associated with illness duration were also found in the overall compliance level (15 points for “under 4 years” vs. 11 points for “4 years or more”) (Table 4).

**Table 4:** Statistics for scores (median, IQR and range) in each RHFCQ domain, broken down by selected clinical variables (EF, duration of illness, symptom severity, and number of comorbidities)–Mann-Whitney U-test results.

Adherence to HF treatment (RHFCQ)	EF		*p*	Duration of illness		*p*	NYHA class		*p*	Comorbidity		*p*	Hospitalization		*p*
	≥45%	<45%		4 years	> 4 years		I or II	III or IV		1	>1		1	> 1	
	N = 327	N = 148		N = 212	N = 263		N = 235	N = 140		N = 240	N = 235		N = 250	N = 225	
1. Follow-up appointment-keeping	4 [3; 4]	3 [2; 4]	**<0.001**	4 [2; 4]	4 [3; 4]	0.097	4 [2; 4]	4 [3; 4]	**0.001**	3 [2; 4]	4 [3; 4]	**0.015**	4 [3; 4]	3 [2; 4]	**<0.001**
	0–4	0–4		0–4	0–4		0–4	0–4		0–4	0–4		0–4	0–4	
2. Taking medication as prescribed	4 [3; 4]	3 [2; 4]	**<0.001**	4 [3; 4]	4 [3; 4]	0.312	4 [3; 4]	4 [3; 4]	**<0.001**	3 [2; 4]	3 [3; 4]	0.913	4 [4; 4]	3 [2; 4]	**<0.001**
	0–4	0–4		0–4	0–4		0–4	0–4		0–4	0–4		0–4	0–4	
3. Daily weighing	0 [0; 2]	0 [0; 1]	0.177	2 [0; 2]	0 [0; 2]	**<0.001**	2 [0; 2]	0 [0; 2]	**0.019**	2 [0; 3]	0 [0; 3]	0.063	0 [0; 3]	0 [0; 3]	0.779
	0–3	0–3		0–3	0–4		0–3	0–4		0–3	0–4		0–4	0–4	
4. Restricted sodium intake	2 [1; 3]	2 [1; 3]	0.741	3 [2; 3]	2 [1; 3]	**<0.001**	3 [2; 3]	2 [1; 3]	0.651	2 [1; 4]	2 [1; 3]	0.153	2 [1; 3]	2 [1; 3]	0.844
	0–4	0–4		0–4	0–4		0–4	0–4		0–4	0–4		0–4	0–4	
5. Restricted fluid intake	2 [0; 3]	2 [1; 3]	**0.021**	2 [1; 3]	1 [0; 3]	**<0.001**	2 [1; 3]	1 [0; 3]	0.744	3 [1; 3]	2 [1; 3]	**0.010**	2 [0; 3]	1 [1; 3]	**0.033**
	0–4	0–4		0–4	0–4		0–4	0–4		0–4	0–4		0–4	0–4	
6. Regular exercise	1 [0; 2]	0 [0; 2]	**0.011**	1 [0; 3]	0 [0; 1]	**<0.001**	1 [0; 3]	0 [0; 1]	**<0.001**	1 [0; 2]	0 [0; 2]	**<0.001**	1 [0; 2]	0 [0; 2]	**<0.001**
	0–4	0–4		0–4	0–4		0–4	0–4		0–4	0–3		0–4	0–4	
7. Healthy lifestyle	6 [3; 10]	8 [3; 10]	0.204	7 [5; 11]	4 [2; 8]	**<0.001**	7 [5; 11]	4 [2; 8]	0.093	7 [5; 11]	6 [3; 9]	**0.002**	6 [2; 11]	6 [3; 9]	0.787
	0–15	0–15		0–16	0–14		0–16	0–14		0–16	0–15		0–16	0–16	
Overall adherence level	13 [9; 17]	13 [9; 17]	0.497	15 [11; 18]	11 [8; 15]	**<0.001**	15 [11; 18]	11 [8; 15]	**0.003**	15 [11; 18]	13 [9; 16]	**0.015**	13 [10; 18]	12 [8; 16]	**0.002**
	0-15	0-15		0-16	0-14		0-16	0-14		0-16	0-15		0-16	0-16	

Median [IQR] and range

Comparisons based on disease symptom severity also showed significant differences, with lower compliance scores obtained by patients in NYHA classes III and IV than by those in classes I and II, in the following domains: “follow-up appointment keeping” (3 vs. 4), “taking medication as prescribed” (2 vs. 4), “daily weighing” (0 vs. 2), “regular exercise” (0 vs. 1), and overall compliance level (11 vs. 15 points).

This comparative analysis also demonstrated differences in compliance based on the number of comorbidities. Patients with HF and up to one comorbidity showed better compliance than those with HF and more than one comorbidity in the following domains: “restricted fluid intake” (3 vs. 2), “regular exercise” (1 vs. 0), and overall compliance level (15 vs. 13 points). In turn, patients with HF and two or more comorbidities showed better compliance than those with one or no HF comorbidities in the “follow-up appointment-keeping” domain (4 vs. 3).

Differences in compliance were also associated with the number of re-hospitalizations due to HF. Patients who had been hospitalized more than once for an HF exacerbation in the past year obtained lower scores than those who had not been hospitalized for exacerbations in the following domains: “follow-up appointment-keeping” (3 vs. 4), “taking medication as prescribed” (3 vs. 4), “restricted fluid intake” (1 vs. 2), “regular exercise”(0 vs. 1) and overall compliance level (12 vs. 13 points).

As recommended by the authors of the original RHFCQ version, patients are considered compliant if they choose “most of the time” or “always” in the follow-up appointment-keeping, restricted sodium intake, restricted fluid intake, and regular exercise domains, and “every day” or “three times a week” in the daily weighing domain [14]. Only 35 such patients were found in the study group (6.9%). These patients were taken to represent the “gold standard” of compliance used in ROC curve analysis to determine cut-off values for continuous and ordinal variables (duration of illness, NYHA class, number of comorbidities, number of hospitalizations). Analysis results are shown in Table 5. The lower limits of the 95% confidence intervals for the area under curve (AUC) are higher than 0.5, indicating that the 4 factors analyzed in Table 5 are statistically significant (Table 5).

**Table 5:** ROC curve analysis results–cut-off values for classification of patients as *compliant* or *non-compliant* for continuous and ordinal variables.

Variable	Cut-off	Sensitivity	Specificity	AUC (95% CI)
Age (years)	> 65	60.6%	68.8%	0.680 (0.636, 0.722)
EF (%)	≤ 45	48.1%	56.5%	0.612 (0.560, 0.664)
Duration of illness (years)	≤ 4	75.8%	57.7%	0.664 (0.619, 0.706)
NYHA class	≤ II	85.7%	38.7%	0.618 (0.567, 0.668)
Number of comorbidities	≤ 1	65.5%	50.9%	0.598 (0.547, 0.648)
Number of hospitalizations	≤ 2	100.0%	34.6%	0.620 (0.574, 0.664)

### Correlation analysis for the impact of selected variables on treatment compliance

In single-factor analysis, negative predictors of compliance in the “follow-up appointment-keeping” domain included: age above 65, living alone, NYHA class III or IV, number of hospitalizations, and treatment with diuretics. Positive predictors of compliance in this domain included: EF (the higher the EF, the higher the compliance score, presence of comorbidities, and treatment with beta-blockers or ACE inhibitors/ARBs (Table 6).

**Table 6:** Simple and multiple regression results.

	Single-factor regression		Multiple-factor regression	
	*b*	*p*	*Beta*	*p*
**Follow-up appointment keeping**				
Age (years)	–0.014	0.014	-	> 0.05
Living alone (yes)	–0.567	<0.001	–0.317	0.010
EF (%)	0.020	<0.001	-	> 0.05
HF symptoms–NYHA class (I, II, III, IV)	–0.344	0.001	-	> 0.05
Number of comorbidities	0.117	0.008	0.123	0.008
Number of hospitalizations	–0.172	<0.001	-	> 0.05
Diuretics (yes)	–0.304	0.007	-	> 0.05
Beta-blockers (yes)	0.365	<0.001	-	> 0.05
Calcium channel blockers (yes)	0.501	<0.001	-	> 0.05
**Taking medication as prescribed**				
Female (yes)	–0.325	<0.001	-	> 0.05
Age (years)	–0.014	0.014	-	> 0.05
Living alone (yes)	–0.559	<0.001	–0.205	0.031
EF (%)	0.020	<0.001	0.009	0.035
HF symptoms–NYHA class (I, II, III, IV)	–0.387	<0.001	–0.181	0.024
Number of hospitalizations	–0.242	<0.001	–0.117	<0.001
Diuretics (yes)	–0.276	0.010	-	> 0.05
Beta blockers (yes)	0.284	0.002	-	> 0.05
Calcium channel blockers (yes)	0.347	0.004	-	> 0.05
**Diet total score**				
Age (years)	–0.126	<0.001	–0.072	<0.001
Living alone (yes)	–2.369	<0.001	–2.039	<0.001
Duration of illness (years)	–0.154	<0.001	-	> 0.05
Number of comorbidities	–0.919	<0.001	–0.676	0.001
Beta-blockers BB (yes)	–1.369	0.001	–0.906	0.025
Calcium channel blockers (yes)	–2.611	0.006	–2.816	0.001
**Overall adherence level**				
Age (years)	–0.165	<0.001	-	> 0.05
Living alone (yes)	–3.002	<0.001	–1.816	0.001
Duration of illness (years)	–0.179	<0.001	-	> 0.05
Symptoms–NYHA class (I, II, III, IV)	–1.610	<0.001	–1.698	<0.001
Number of comorbidities	–0.838	0.001	–0.489	0.021
Number of hospitalizations	–0.729	<0.001	-	> 0.05
Beta blockers (yes)	–1.612	0.001	-	> 0.05
Calcium channel blockers CCBs (yes)	–2.120	0.001	–2.319	0.027

In the “taking medication as prescribed”, compliance was negatively associated with: female gender, age above 65, living alone, NYHA class > II, more than one hospitalization, and treatment with diuretics. Positive predictors of compliance in this domain were EF<45% and treatment with beta-blockers or ACE inhibitors/ARBs.

In the healthy lifestyle domain (combined score), compliance was negatively associated with: age above 65, living alone, duration of illness above 4 years, two or more comorbidities, and treatment with blockers or ACE inhibitors/ARBs.

Negative predictors of overall compliance level in single-factor analysis were: age above 65, living alone, duration of illness above 4 years, NYHA class III or IV, more than one comorbidity, more than one hospitalization, and treatment with blockers or ACE inhibitors/ARBs.

To verify which of the variables analyzed are independent predictors, multiple-factor analysis was performed. In the “follow-up appointment-keeping” domain, living alone was a negative independent predictor, while the number of hospitalizations due to HF was a positive independent predictor. In the “taking medication as prescribed” domain, negative independent predictors were: living alone, NYHA class, and number of hospitalizations. EF was an independent predictor of higher compliance in the domain.

In the healthy lifestyle domain (combined score for fluid and sodium restriction and weighing), negative independent predictors of compliance were: living alone; age > 65 years, number of comorbidities, and treatment with beta-blockers or ACE inhibitors/ARBs.

With regard to the overall compliance level, negative independent predictors were: living alone, NYHA class, number of comorbidities, and treatment with ACE inhibitors/ARBs.

Based on the overall compliance scores, if patients who scored below 18 points for the 6 items are assumed to be non-compliant, odds ratios can then be calculated.

The odds of non-compliance with treatment in patients aged above 65 are nearly two-and-a-half times as high as in patients aged 65 or younger (OR = 2.47) (Table 7). The odds are even higher in patients living alone (OR = 2.60), those having lived with HF for more than 4 years (OR = 2.81), and those with NYHA class higher than II (OR = 2.19). An increased risk was also found in patients with comorbidities (1.79) and those having had multiple re-hospitalizations (2.07). Based on the oral medications taken, the odds of non-compliance were higher by 1.80 in patients treated with beta-blockers, 1.74 in those treated with diuretics, and 1.57 in those treated with ACE inhibitors/ARBs. The odds for ACEi/ARBs were not statistically significant.

**Table 7:** Multiple logistic regression analysis for the discrimination of non-compliance.

Risk factor	Cut-off value	Non-compliance < 18 N = 407		Compliance >18 N = 68		P	OR (95% CI)
		n	%	n	%		
Female gender	Yes	187	45.9	35	51.5	0.510	0.80 (0.48, 1.34)
Age (years)	> 65	290	71.3	27	39.7	<0.001	2.47 (1.44, 4.22)
Living alone	Yes	189	46.4	17	25.0	0.001	2.60 (1.45, 4.66)
Duration of illness (years)	> 4	240	59.0	23	33.8	<0.001	2.81 (1.64, 4.82)
Symptoms–NYHA class	> 2	129	34.9	11	22.9	0.027	2.19 (1.08, 4.55)
Number of comorbidities	> 1	165	51.7	21	37.5	0.050	1.79 (1.00, 3.20)
Number of hospitalizations	> 2	203	49.9	22	32.4	0.007	2.07 (1.21, 3.58)
Diuretics	Yes	335	82.3	49	72.1	0.047	1.80 (1.00, 3.25)
Beta-blockers	Yes	285	70.0	39	57.4	0.038	1.74 (1.03, 2.94)
ACE inhibitors/ARBs	Yes	62	15.2	7	10.3	0.285	1.57 (0.68, 3.58)

*p*–significance in the Pearson chi-squared test, OR–odds ratio, 95% CI– 95% confidence interval for the odds ratio

## Discussion

Compliance with pharmacological treatment, as well as to the recommended lifestyle changes, is a key to the successful treatment of chronic disease. An understanding of factors determining compliance with pharmacological and non-pharmacological HF treatment is required for adequate treatment planning.

The present findings is one of a few studies, which provide information firstly on compliance with pharmacological and non-pharmacological treatment among eldery patients with HF, secondly it should be underlined that the analysis of the compliance of pharmacological and nonpharmacological recommendation of life style modifications. The poorest compliance levels were found for exercise, daily weighing, and restricted sodium intake. In these areas, compliance was found in one in three or even one in five patients.

Multiple authors have described problems with compliance with a low-sodium diet. In a study by Riegel et al., patients scored particularly low in terms of a low-salt diet, and the authors reported that the problem often resulted from a misinterpretation or misunderstanding of the prescribed restrictions [15]. Many patients following a diet do not pay attention to the sodium content in each product that they eat. Similar findings were reported in a study by Colin-Ramirez et al., where 4% of patients declaring that they always follow the sodium intake restrictions still eat processed foods with a high sodium chloride content, as they interpreted the restriction as only applicable to their use of table salt when preparing their own meals [16]. One must also bear in mind the difficulties involved in modifying habits that had been formed at a very early stage of life [17]. Van der Wal et al. have emphasized the problem of non-compliance with salt and fluid restrictions [14,18].

In previous studies, similarly low scores were obtained for compliance with daily weighing and exercise [19]. However, in the present study, the level of compliance with these recommendations was shockingly low. As few as 4% of patients “always” exercised as ordered, and 13.5% did it “most of the time”. In other studies, the mean level of non-compliance with exercise recommendations was 40% [20]. Some forms of non-compliance may be due to a lack of clear information and recommendations from medical staff, associated with a lack of an appropriate out-of-hospital care system for HF patients in a given population. However, key factors that are likely to affect the entire patient population also include poor motivation and insufficient knowledge of the benefits of regular exercise [21].

In the present study, the level of compliance with the recommended fluid restriction was also very low. According to Riegel et al., lack of fluid restriction in patients’ diets may be explained by difficulties in the precise determination of one’s fluid intake, as well as not testing for thirst levels, which may be significant to this area of compliance [15].

The present study has demonstrated that nearly 60% of patients reported that they always took their medication as prescribed, which corroborates findings presented in the literature [22,23]. Most differences in compliance levels were associated with the use of diuretics and beta-blockers, but reports on compliance with diuretic treatment are contradictory. In the present study, patients treated with diuretics obtained the lowest compliance scores, and treatment with diuretics was associated with the highest odds of pharmacological non-compliance as well as nonpharmacological treatment, which comprises diet, weighing, and restricted salt and fluid intake. Some authors reported high compliance rate in this respect [15,22,24], for instance Riegel et al. reported a surprisingly high r odds ratios. In the literature, the discussion on the associations between men and women and compliance rate to the diuretic regimen (84.7% of patients) [15].

It is possible that the discomfort associated with increased diuresis causes patients to take lower or irregular doses and, as a secondary consequence, to intentionally miss their follow-up appointments. This results in non-optimal compensation of high volemia, increased risk of cardiovascular decompensation, and poorer prognosis, which in turn necessitates a higher dosage of diuretics due to disease exacerbation and increased fluid retention, producing a vicious cycle [25]. In the present study, treatment with beta-blockers and ACE inhibitors or ARBs correlated positively with pharmaceutical compliance and regular follow-up appointment-keeping, but a negatively one on the overall compliance level and healthy lifestyle. Patients must see their physician often to renew their prescription, but may assume that pharmacological treatment alone is sufficient or more important than lifestyle changes. Cottrell et al. demonstrated that patients who believed their ACE inhibitor, ARB or beta-blocker was an important and necessary part of their treatment disregarded the importance of salt and fluid restriction. Some patients tend to think that compliance with pharmacological treatment can prevent health deterioration and negative outcomes, even if they do not comply with the required lifestyle changes [26,27].

Other determinants of treatment compliance include patient- and health-related factors. Single-factor regression analysis performed in the present study demonstrated a negative impact of female gender on pharmacological compliance and follow-up appointment keeping, whereas healthy lifestyle and overall compliance scores were not associated with a low level of compliance. Gender was not an independent predictor of compliance in multiple regression analysis and had no significance fo with treatment in cardiovascular disease is still ongoing [28–30]. There are few publications that discuss the impact of gender on compliance in HF, but those that do show a positive impact for male patients [3,31–33]. The question remains as to whether men actually comply with treatment better than women, or whether this effect results from women’s support and involvement in care for their family members.

Another factor that is often discussed as a predictor of compliance with HF treatment is older age [26, 30]. Older age, cognitive impairment, or frailty syndrome are often predictors of significantly poorer compliance with pharmacological treatment and lifestyle changes [31]. The present study corroborates previously published reports [13,31,34]. Older age was a negative predictor of follow-up appointment-keeping, medication-taking, lifestyle changes, and overall compliance level. The most common reason for lower compliance among older age patients is forgetfulness, which has been recognized as a crucial barrier to medication compliance [35–38]. Old age is associated with progressive cognitive impairment. Older patients also have more comorbidities that require compliance with complex treatment protocols, which may be difficult for the elderly. Therefore, simplifying treatment protocols in this patient group might seem clinically reasonable, especially in case of polypharmacy, multiple morbidities and treatment by many specialists. The good solution to nonadherence in elderly is the applying of the systems amplifying the level of adherence (pill boxes or electronic bells), the developing habits of taking medicines to combine them with activities performed at fixed times (rituals). The visual reminders in home environment are very useful as well as the guidance and education adjusted to physical and cognitive possibilities of patients and the social support.

It should be underlined that among socio-demographic factors, social support and loneliness are often negative predictors of compliance, especially among older age patients. In the present study, living alone was an independent determinant of follow-up appointment keeping, medication-taking, and lifestyle changes, and overall compliance scores. The role of social support provided by family members and other informal caregivers in the care over HF patients is emphasized as a factor which positively affects compliance [39–41]. Family members often support the patient in developing a habit of taking medication, as well as in getting to follow-up tests and visits [41].

Predictors of compliance also include clinical factors [23,42]. In the present study, patients who had had HF for more than 4 years, were classified in NYHA class II or higher, had comorbidities, and had undergone multiple hospitalizations were more likely to be non-compliant. Higher NYHA classes and a higher number of comorbidities were independent determinants of lower overall compliance levels. However, one must bear in mind that the association observed does not necessarily indicate the direction of causality. Patients who do not fully comply with treatment tend to have more severe disease symptoms. The present study contributes to the ongoing discussion on disease severity and symptoms as determinants of compliance. Rockwell and Riegel [43] found that patients who were symptomatic were more likely to take their medication as prescribed. In turn, according to van der Wal and Jaarsma, patients in NYHA classes III and IV with an EF of ≤40% reported significantly more barriers and fewer benefits in compliance with treatment. The authors point to depression and a pessimistic attitude towards life, often found in HF patients, as factors that may considerably affect treatment compliance [14, 27].

## Study limitations

Our study has several limitations. One limitation of the present study was the use of a self-reported questionnaire, as patients may have overestimated their compliance in an attempt to give socially desirable answers. Furthermore, with regard to compliance with the recommended restrictions of salt and fluid intake, we did not use any measure of actual intake. Difficulties in evaluating the causal relationship between compliance with pharmacological and non-pharmacological treatment and disease symptoms may also be significant, as discussed in the article. In the present study, compliance with fluid intake restrictions was only analyzed based on patients’ self-report on restriction of fluid intake in the past week, with no specific quantification. An additional limitation of the study is the use of a questionnaire that has good psychometric properties, but the validation process has not yet been published. Heart failure occurs primarily in the group of elderly patients. Unfortunately, on the other hand, elderly patients are not represented in large numbers in available studies. That is why we decided to include this group as representative of the studied problem. Unfortunately, there is still a lack of strong evidence about the level of adherence also in younger groups of patients with HF. Therefore, comparing elderly people with younger people can be an interesting topic for continuing further research. The lack of such comparisons may be another limitation of our study.

## Conclusions

1. Patients with HF do not fully adhere to chronic treatment. Compliance with pharmacological and non-pharmacological HF treatment differs. The lowest compliance levels were found for exercise and daily weighing, and the highest for follow-up appointment-keeping and medication.
2. Socio-clinical factors: older age and living alone, as well as clinical ones: duration of illness, higher NYHA classes, and treatment with diuretics, were predictors of compliance.
3. The findings of the present study provide evidence to physicians and healthcare policymakers that compliance with treatment among HF patients is low, and that there is no single factor that can be deemed solely responsible for shaping treatment compliance in these patients, as the problem of non-compliance is multidimensional.

## Implications for practice

Daily medical practice should include continuous evaluation of compliance with HF treatment, so that non-adhering patients can receive additional attention. Patients need information to understand the nature of their illness and the importance of compliance with treatment. Each visit should be accompanied by counseling in order to improve compliance with treatment and the patient’s perception of the illness. Healthcare providers need to focus on patients’ behaviors that may interfere with compliance with treatment in order to achieve control of HF in the community. Healthcare professionals should identify potential viable strategies for increasing compliance in their daily practice. The role of family members and caregivers in enhancing compliance with therapeutic recommendations should be emphasized.
